# Beyond the Acknowledged Limitations: Critical Methodological Gaps in Prognostic Assessment of Symptom Burden Among Terminal Cancer Patients

**DOI:** 10.1002/jgf2.70125

**Published:** 2026-05-14

**Authors:** Shaher Yar, Aiman Aijaz, Khadija Sana, Ishvah Shashwat Nag

**Affiliations:** ^1^ University College of Medicine and Dentistry Lahore Pakistan; ^2^ State Non‐Profit Enterprise Danylo Halytsky Lviv National Medical University Lviv Ukraine

To the Editor,

We read with considerable interest the recent study by Bussaba and colleagues, which examined survival differences between high and low symptom burden, as measured via the Edmonton Symptom Assessment System (ESAS), among terminal cancer patients with Palliative Performance Scale scores of 30 or below [1]. Their use of restricted mean survival time (RMST) to express survival disparities in tangible days meaningfully enhances clinical interpretability, and the reported 3.7‐day survival reduction associated with high physical symptom burden carries direct relevance for end‐of‐life care. Several methodological gaps, however, warrant scrutiny before these findings enter broader prognostic frameworks.

The study dichotomizes ESAS scores into high (≥ 4) and low (< 4) categories using a threshold borrowed from prior literature without validation in this terminal cohort. Collapsing a continuous 10‐point scale into binary strata discards statistical information and assumes an abrupt mortality transition at the cutoff rather than a gradual dose–response relationship [2]. A dose–response analysis using restricted cubic splines would clarify whether mortality risk accumulates linearly or concentrates at extreme values, strengthening the biological plausibility of the reported association.

Although the authors acknowledge unmeasured confounding, noting medication use in a limitations paragraph is categorically distinct from incorporating it as a model covariate. Opioids and benzodiazepines, routinely prescribed for pain and dyspnea in this population, can simultaneously suppress symptom scores while influencing survival through dose‐dependent respiratory and sedative effects [3]. A patient recording a low ESAS score may not be inherently stable; they may be pharmacologically suppressed. Adjusting for oral morphine equivalent daily dose (OMEDD) or sedation status, data routinely documented in palliative records, would disentangle true symptom burden from medication‐mediated confounding.

The Emotional Symptom Subscale (ESS) not only failed to reach statistical significance as a standalone predictor but also did not retain significance in multivariable models, where only the physical domain emerged as an independent predictor of survival. Before attributing this to absent prognostic value, the two‐item composition of the ESS warrants scrutiny [4]. Validated instruments, such as the Hospital Anxiety and Depression Scale (HADS) or PHQ‐9, offer greater construct validity and have demonstrated independent prognostic significance in advanced cancer populations. Their use in future analyses would clarify whether this null finding reflects biology or measurement.

The cohort encompassed multiple heterogeneous primary cancer types, including gastrointestinal, lung, and hepatobiliary malignancies, each with distinct symptom profiles and survival trajectories. No subgroup analysis stratified by cancer type was conducted, nor were interaction terms tested [5]. The authors themselves recommend such analyses in future work, implicitly conceding that cancer‐type heterogeneity limits the present findings, yet the data required for stratification existed within their cohort and were not utilized. Pooling heterogeneous malignancies without exploring effect modification risks obscures cancer‐specific signals or amplifies associations driven by a single tumor subtype.

Addressing these concerns—continuous variable modeling, pharmacological covariate adjustment, validated psychological instruments, and cancer‐type stratification—would substantially strengthen the evidentiary foundation of this well‐conceived investigation. We commend the authors and encourage these refinements in future multicenter work.

## Author Contributions

**Shaher Yar:** conceptualization, writing – original draft. **Aiman Aijaz:** writing – original draft. **Khadija Sana:** writing – review and editing. **Ishvah Shashwat Nag:** project administration, writing – review and editing.

## Funding

The authors have nothing to report.

## Conflicts of Interest

The authors declare no conflicts of interest.

## Data Availability

The authors have nothing to report.
